# Medical History of the Expedition to the Niger

**Published:** 1843-09-30

**Authors:** 


*Medical History of the Expedition to the Niger, during the years* 1841–42, *comprising an Account of the Fever which led to its abrupt Termination.* By James Ormiston McWilliam, M. D., Senior Medical Officer of the Expedition, &c. London: 1843.

